# Differences in retroactive interference response reveal sex-specific memory resilience in pre-symptomatic triple transgenic Alzheimer’s disease mice

**DOI:** 10.1093/braincomms/fcag345

**Published:** 2026-09-11

**Authors:** Chiara D’Amelio, Sara Autelitano, Francesca Natale, Marco Rinaudo, Salvatore Fusco, Claudio Grassi

**Affiliations:** Department of Neuroscience, Università Cattolica del Sacro Cuore, Rome 00168, Italy; Department of Neuroscience, Università Cattolica del Sacro Cuore, Rome 00168, Italy; Department of Neuroscience, Università Cattolica del Sacro Cuore, Rome 00168, Italy; Fondazione Policlinico Universitario ‘A. Gemelli’ IRCCS, Rome 00168, Italy; Department of Neuroscience, Università Cattolica del Sacro Cuore, Rome 00168, Italy; Fondazione Policlinico Universitario ‘A. Gemelli’ IRCCS, Rome 00168, Italy; Department of Neuroscience, Università Cattolica del Sacro Cuore, Rome 00168, Italy; Fondazione Policlinico Universitario ‘A. Gemelli’ IRCCS, Rome 00168, Italy; Department of Neuroscience, Università Cattolica del Sacro Cuore, Rome 00168, Italy; Fondazione Policlinico Universitario ‘A. Gemelli’ IRCCS, Rome 00168, Italy

**Keywords:** Alzheimer’s disease, memory, learning, interference, sex

## Abstract

Alzheimer’s disease is a neurodegenerative disorder characterized by memory loss dependent on hippocampal and parahippocampal cortex dysfunction. However, recent evidence shows that patients with mild cognitive impairment progressing to Alzheimer’s disease can exhibit early symptoms when assessed with more nuanced memory tests, such as those evaluating memory vulnerability to interference. Here, we characterize how sex and genotype shape the response to retroactive interference in triple-transgenic Alzheimer’s disease mice at an early stage of the phenotype, when overt memory deficits are not yet present. Wild-type females were the only group resisting interference: transgenic females failed to discriminate despite normal recognition memory, irrespective of the moment during which retroactive interference was experienced, and males of both genotypes were similarly impaired at every time point tested. Both context change and increased memory strength restored discrimination in transgenic females and wild-type males, suggesting that their susceptibility depends on overlapping hippocampal representations. Transgenic males, by contrast, remained impaired when interference occurred in a different context and showed only a limited improvement after multiple training, pointing to a larger contribution of interference-induced memory decay. Overall, our data characterize the response to retroactive interference as a sex-dependent behavioural readout of early memory instability in a preclinical model of Alzheimer’s disease.

## Teaser

D’Amelio *et al*. report that, in a triple-transgenic Alzheimer’s disease mouse model, susceptibility to retroactive interference reveals early, sex-specific memory fragility before overt deficits appear: transgenic females lose the interference resilience shown by wild-type females, while transgenic males show a more persistent, harder-to-rescue impairment.

## Graphical Abstract

**Graphical Abstract:**
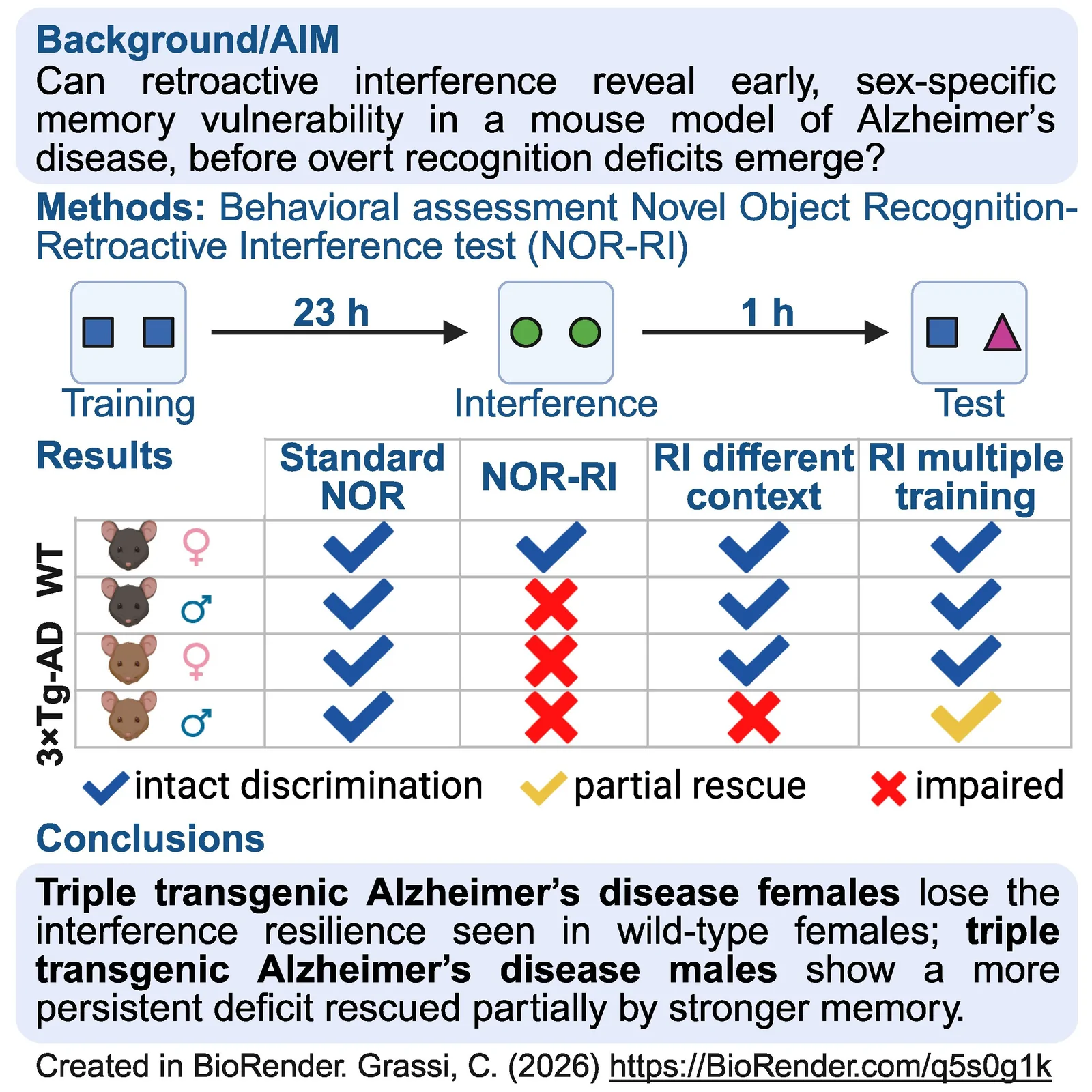

## Introduction

Alzheimer’s disease is a neurodegenerative disorder characterized by memory impairment and a progressive loss of executive functions,^1^ which dramatically and irreversibly hinders an individual’s quality of life. Recent findings indicate that Alzheimer’s disease pathological hallmarks, such as amyloid-β (Aβ) and tau proteins, accumulate years before the emergence of overt cognitive impairment.^2,3^ Aβ deposition and tau pathology are detectable in clinically unimpaired individuals, yet standard cognitive screening tools such as the Montreal Cognitive Assessment (MoCA) and Mini-Mental State Examination (MMSE) often remain within normal ranges during these early stages.^4,5^ This dissociation highlights a critical gap: the lack of sensitive cognitive markers capable of revealing subtle network instability before global memory alterations become clinically evident.

Emerging human evidence suggests that interference-based paradigms may function as cognitive ‘stress tests’ capable of unmasking early vulnerability. Tasks that probe susceptibility to proactive or retroactive interference (RI), such as semantic interference paradigms, reveal abnormalities in individuals with mild cognitive impairment (MCI) and even in cognitively normal adults with Aβ positivity.^6,7^ Interference vulnerability is dependent on the interaction between different brain structures, such as the prefrontal cortex, the hippocampus and associated cortices.^8-11^ Multiple mechanisms have been identified in regulating interference, such as dopaminergic transmission, cytoskeletal control of synaptic plasticity and sex hormone-related signalling at the hippocampal level,^12-14^ all of which have been observed to be dysregulated in MCI and Alzheimer’s disease models.^15^ However, despite growing clinical interest in interference paradigms, several fundamental questions regarding interference vulnerability, such as whether vulnerability/resilience to RI is sex-specific, remain unanswered.

Sex differences in interference susceptibility are likely to reflect the organization of hippocampal oestrogenic signalling. Oestrogen receptor α (ERα) is expressed throughout the dorsal hippocampus, where it couples to extracellular signal-regulated kinase (ERK1/2) signalling to support memory consolidation in females.^16^ The oestradiol-engaged cascade also differs between the sexes: in male mice, intrahippocampal oestradiol enhances memory consolidation without increasing ERK1/2 phosphorylation, and its effect is insensitive to ERK inhibition.^17^ Sex differences extend to the dentate gyrus, where adult-born neurons mature faster and undergo greater attrition in males than in females,^18^ with potential consequences for the separation of similar representations. Therefore, sex-specific hippocampal circuitry and molecular cascades control how memories are stored and retrieved in the male and female hippocampus.

However, the extent of memory vulnerability to RI remains poorly characterized, particularly in the context of Alzheimer’s disease. To this end, we performed a series of tests to assess the boundaries of memory vulnerability in male and female WT and triple-transgenic Alzheimer’s disease (3×Tg-AD) mice by manipulating different parameters of interference, such as timing, the context in which interference happens, and the strength of the memory subjected to interference. Building on previously published works showing that WT female mice are resistant to RI whereas males are not,^19^ we show here that female 3×Tg-AD mice lose this resilience, reaching a level of performance comparable to that of males of either genotype. This loss is not restricted to a specific temporal window, as RI performed during or after consolidation, as well as before memory retrieval, impaired object recognition memory in transgenic females, and the same was observed in WT and transgenic males at every time point tested. Moreover, context switching during RI and increased memory strength both restored the discrimination index (DI) in female 3×Tg-AD mice as well as in WT males, whereas male 3×Tg-AD mice were not rescued by context change and improved only partially after multiple training. Collectively, our results indicate that early AD-related pathology abolishes a female-specific resilience to interference and that the residual deficit is differently tractable in the two sexes.

## Materials and methods

### Animals

Male and female WT C57BL/6JRj and 3×Tg-AD mice (2 months old) obtained from the Animal Facility of the Catholic University were used for this study. Animals were housed under standard laboratory conditions with a 12 h light/dark cycle, with constant humidity (60–75%), controlled room temperature (20°C–22°C) and food and water provided *ad libitum*.

### Ethics

All experiments and animal procedures were approved by the Catholic University Ethics Committee and were in accordance with the guidelines of the Italian Ministry of Health (Legislative Decree No. 116/1992) and European Union legislations on animal procedures (Directive No. 86/609/EEC) and were approved by the Italian Ministry of Health, procedure number 312/2026-PR.

### Behavioural analyses

Behavioural analyses were performed from 9 a.m. to 4 p.m. by an experimenter blind to the treatments. The data were blindly analysed using the behavioural tracking system ANY-Maze^TM^ (Stoelting Co., Wood Dale, IL, USA). Exploration time (defined as the time the animal’s snout was directed at the object from <2 cm) was recorded for the novel and the familiar object. The preference index (PI) for the novel and for the familiar object was calculated respectively, as the percentage of time spent exploring the novel object or the familiar object compared to the total object exploration. The DI for the novel object was calculated as follows: (novel object exploration time − familiar object exploration time)/(novel object exploration time + familiar object exploration time). The objects used for the different object recognition paradigms were: Lego cubes, glass bottles, 50 ml-falcon and flasks for cell culture filled with clean bedding. All objects were of similar size. Exploration time from all phases of all experiments is reported in the Supplementary Materials (see Supplementary Table 1).

### Standard novel object recognition paradigm

The standard novel object recognition (NOR) test was used as previously described.^20^ On the first day, the mice were individually subjected to a 10-min habituation session in an empty arena (33 × 33 cm, transparent plexiglass) to familiarize themselves with the apparatus. On the second day, training was conducted in a single 5-min session, where two identical objects were placed symmetrically in the arena. On the third day, during the test phase, one of the familiar objects was replaced with a novel object, and the mice were allowed to explore for 5 min. To further exclude place preference for one side of the arena, the position of the novel object was alternated on both sides during the test session. The objects and arena were cleaned with a 70% ethanol solution for subsequent tests.

### Retroactive interference paradigm

**Figure 1:**
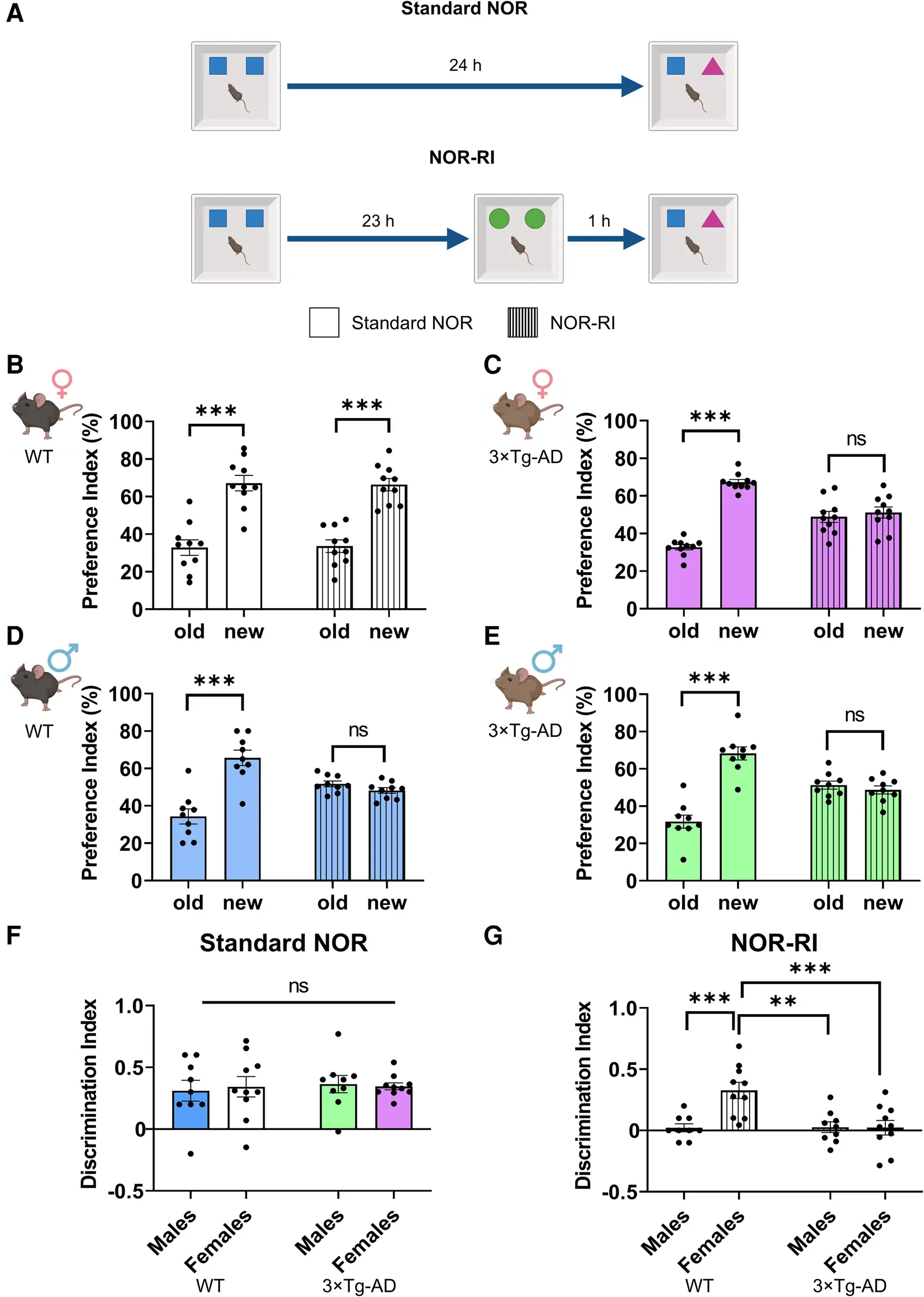
**Female 3×Tg-AD mice show vulnerability to RI-induced memory loss.** (**A**) Schematic representation of the standard NOR task and the NOR-RI variant. (**B–E**) Both male and female WT and 3×Tg-AD mice showed normal recognition memory in standard NOR as evidenced by a significant difference in the PI between novel and old object and no difference in the overall DI, while WT female were the only group showing preserved recognition memory in the RI condition. (**F, G**) Direct comparison of the DI reveals no differences in the standard NOR procedure, with female WT mice being instead the only group discriminating the novel from the old object in the RI paradigm, while 3×Tg-AD female mice failed to discriminate the novel from the familiar object. Standard NOR (**F**) genotype *F*(1,34) = 0.164, *P* = 0.687; sex *F*(1,34) = 0.009, *P* = 0.923; interaction *F*(1,34) = 0.134, *P* = 0.716 (all ns). NOR-RI (**G**): genotype *F*(1,34) = 7.870, *P* = 0.006; sex *F*(1,34) = 7.920, *P* = 0.008; interaction *F*(1,34) = 8.343, *P* = 0.006. Data are presented as mean ± SEM; **P < 0.01; ***P < 0.001; ns: not significant. Student’s *t-*test and two-way ANOVA followed by Tukey’s *post hoc*. Each dot represents an individual animal (*n* = 9–10 mice/group). Panel **A** created in BioRender. Grassi, C. (2026) https://BioRender.com/wf8xzgo.

For the NOR-RI paradigm^19,21^ animals underwent the same standard NOR procedure with the addition of an interference session held 23 h after the training phase and 1 h before the testing phase. During the interference session, two identical objects unrelated to the training objects were placed symmetrically in the middle of the arena, and animals were allowed to explore them for 5 min. One hour later, to evaluate the effect of RI, animals were presented one object from the initial training phase along with a totally novel object, different from the objects of both training and interference, and they were allowed to explore them for 5 min (Fig. 1A).

### Time-based boundaries of RI paradigm

**Figure 2:**
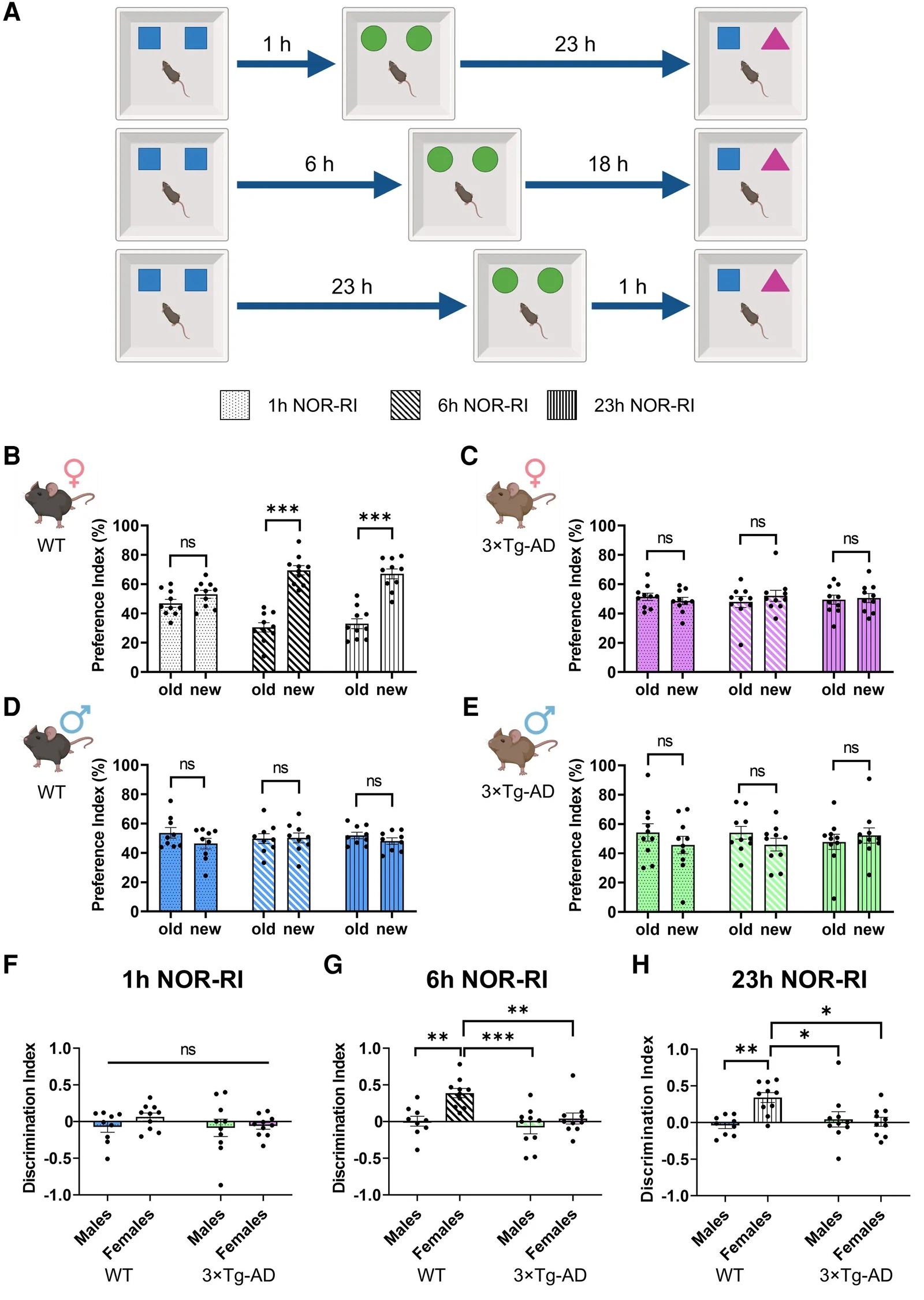
**3×Tg-AD female and male mice and male WT mice are vulnerable to RI-induced forgetting independently from RI timing.** (**A**) Schematic representation of time-based boundaries in NOR-RI paradigm. (**B**) PI in female WT for the old and novel object at 1, 6 and 23 h RI. Female WT mice show deficits in recognition memory only when RI is experienced during memory consolidation, while no deficits are observed at 6 and 23 h RI. (**C**) PI for the old and novel object for female 3×Tg-AD mice. Independently from time of RI exposure, transgenic female mice failed to discriminate the novel object. (**D**) PI in male WT for the old and novel object at 1, 6 and 23 h RI. Male WT mice show deficits in recognition memory independently from time of RI exposure. (**E**) PI for the old and novel object for male 3×Tg-AD mice. Independently from time of RI exposure, transgenic male mice failed to discriminate the novel object. (**F–H**) Comparison of DI for all phases between WT and 3×Tg-AD mice. DI indicates that both WT and 3×Tg-AD male and female mice didn’t discriminate between novel and familiar objects when RI was introduced 1 h after training (**F**), while only female WT mice discriminate the novel from the old object when RI occurred 6 h after training (**G**) and when RI occurred 23 h after training (**H**). 1 h NOR-RI (**F**): genotype *F*(1,35) = 0.717, *P* = 0.402; sex *F*(1,35) = 1.072, *P* = 0.307; interaction *F*(1,35) = 0.476, *P* = 0.494 (all ns). 6 h NOR-RI (**G**): genotype *F*(1,35) = 8.56, *P* = 0.006; sex *F*(1,35) = 11.75, *P* = 0.002; interaction *F*(1,35) = 3.122, *P* = 0.085. 23 h NOR-RI (**H**): genotype *F*(1,35) = 2.765, *P* = 0.105; sex *F*(1,35) = 5.460, *P* = 0.025; interaction *F*(1,35) = 7.701, *P* = 0.009. Data are presented as mean ± SEM; *P < 0.05; **P < 0.01; ***P < 0.001; ns: not significant. Student’s *t-*test and two-way ANOVA followed by Tukey’s *post hoc*. Each dot represents an individual animal (*n* = 9–10 mice/group). Panel **A** created in BioRender. Grassi, C. (2026) https://BioRender.com/ktblcxu.

Animals underwent the same standard NOR procedure with the addition of an interference session held 1, 6 or 23 h after the training phase. The testing phase was performed respectively 23, 18 or 1 h after the interference session (Fig. 2A). The 1 h time point was selected to represent RI during the consolidation phase,^21-23^ the 6 h as a time point outside the early consolidation phase,^22^ and the 23 h as a pre-retrieval time point.^19^

### Retroactive interference in a different context paradigm

**Figure 3:**
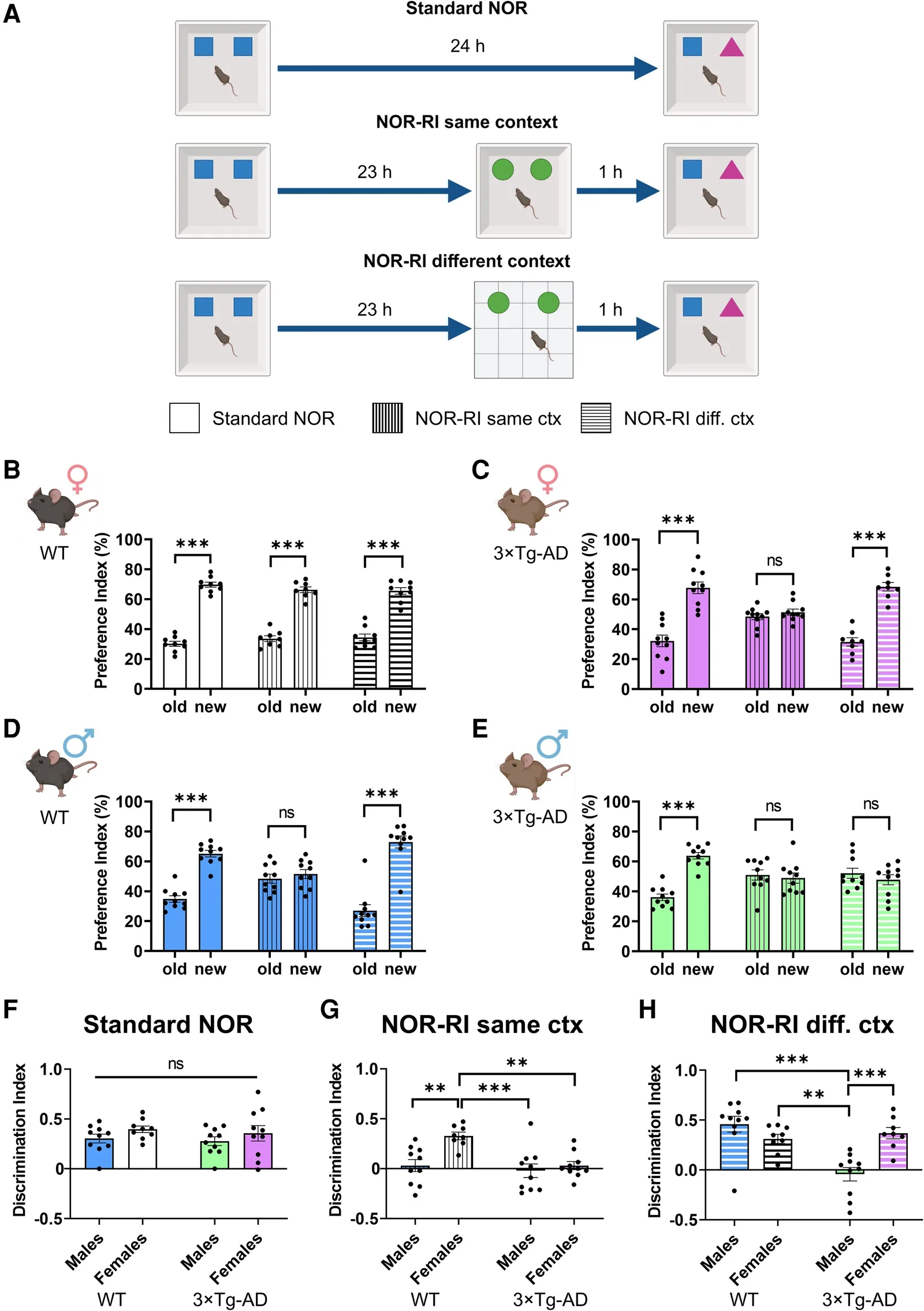
**Context-dependent modulation of RI halts memory deficits in female 3×Tg-AD and male WT but not in male 3×Tg-AD mice.** (**A**) Schematic overview of the NOR-RI conducted in a different context. (**B**) PI for old and novel object in WT female mice. (**C**) PI for old and novel object for 3×Tg-AD female mice, which successfully discriminated between novel and familiar objects when RI was presented in a novel context. (**D**) PI for old and novel object in WT male mice. (**E**) PI for old and novel object in 3×Tg-AD male mice, showing impaired memory even if the NOR-RI was experienced in a different context. (**F–H**) DI compared across sex and genotype across standard NOR (**F**), NOR-RI in the same context (**G**), and NOR-RI in a different context (**H**). Standard NOR (**F**): genotype *F*(1,35) = 0.385, *P* = 0.538; sex *F*(1,35) = 2.592, *P* = 0.116; interaction *F*(1,35) = 0.016, *P* = 0.898 (all ns). NOR-RI same context (**G**): genotype *F*(1,34) = 10.12, *P* = 0.003; sex *F*(1,34) = 10.04, *P* = 0.003; interaction *F*(1,34) = 5.028, *P* = 0.032. NOR-RI different context (**H**): genotype *F*(1,33) = 11.38, *P* = 0.002; sex *F*(1,33) = 4.018, *P* = 0.053; interaction *F*(1,33) = 18.02, *P* < 0.001. Data are presented as mean ± SEM; **P < 0.01; ***P < 0.001; ns: not significant. Student’s *t-*test and two-way ANOVA followed by Tukey’s *post hoc*. Each dot represents an individual animal (*n* = 8–10 mice/group). Same ctx: same context; diff. ctx: different context. Panel **A** created in BioRender. Grassi, C. (2026) https://BioRender.com/bcgztpb.

For the NOR-RI paradigm in a different context^19^ animals underwent the same NOR-RI procedure, however, the interference session was conducted in a different arena (45 × 45 cm, white plexiglass) located in another room from the one used for the training session (Fig. 3A).

### Multiple training RI paradigm

**Figure 4:**
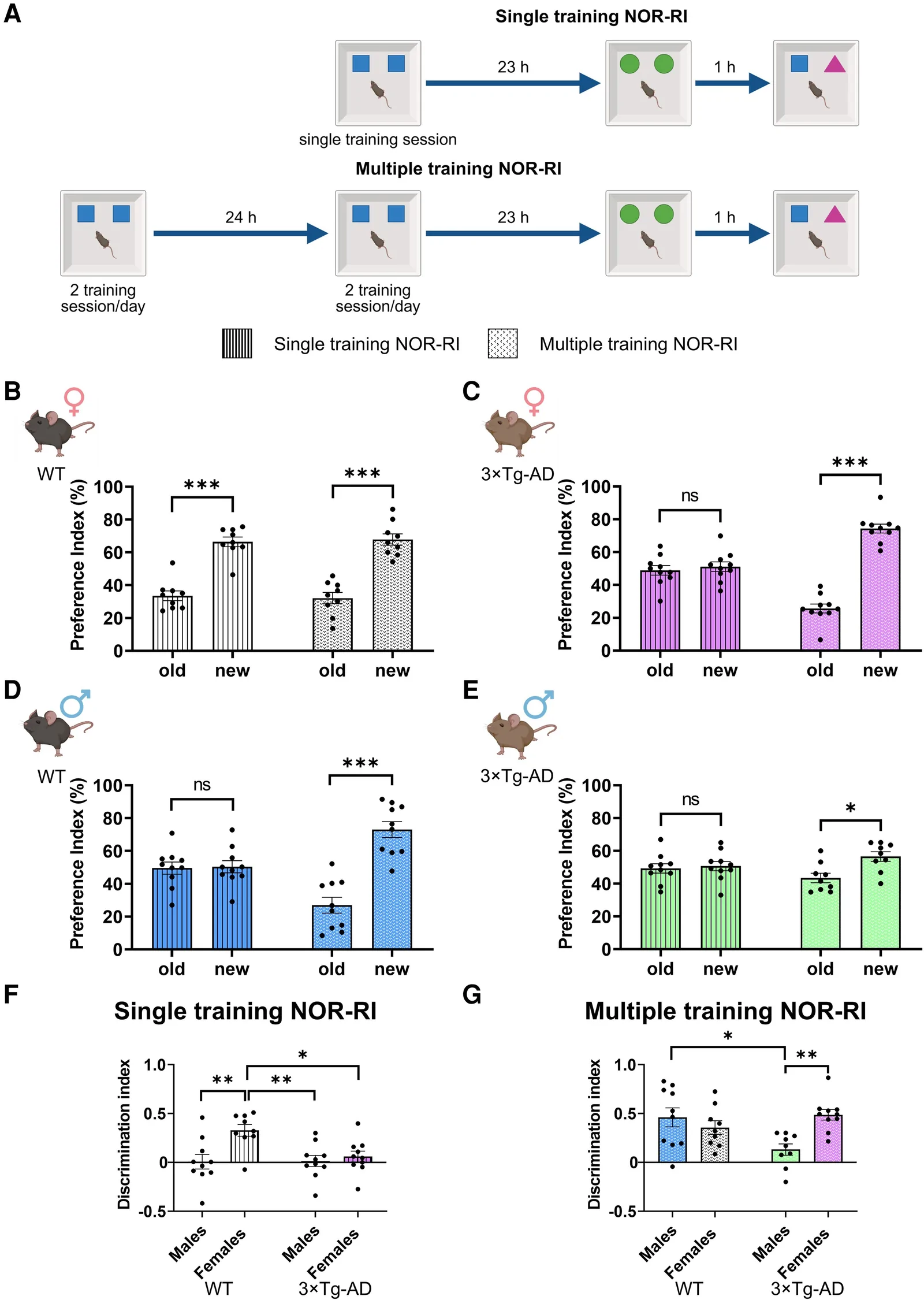
**Interference vulnerability is dependent on memory strength.** (**A**) Schematic illustration of the multiple training NOR-RI paradigm. (**B**) PI for old and novel object for WT female mice. (**C**) PI for old and novel object for 3×Tg-AD female mice showing preserved memory in the multiple training session paradigm. (**D**) PI for old and novel object for WT male mice, which were able to discriminate the novel from the old object in the test phase, if they underwent a multiple training session paradigm before being subjected to the NOR-RI protocol. (**E**) PI for old and novel object for 3×Tg-AD male, showing a significant preference for the novel object. (**F**) DI indicates that both WT and 3×Tg-AD males and 3×Tg-AD female mice showed impaired recognition memory in single training NOR-RI. (**G**) DI indicates that WT males and 3×Tg-AD females have a greater discrimination compared to 3×Tg-AD males, while showing no significant differences from WT females. Single training NOR-RI (**F**): genotype *F*(1,35) = 4.418, *P* = 0.043; sex *F*(1,35) = 8.665, *P* = 0.006; interaction *F*(1,35) = 4.890, *P* = 0.034. Multiple training NOR-RI (**G**): genotype *F*(1,34) = 1.876, *P* = 0.179; sex *F*(1,34) = 2.988, *P* = 0.093; interaction *F*(1,34) = 10.01, *P* = 0.003. Data are presented as mean ± SEM; *P < 0.05; **P < 0.01; ***P < 0.001; ns: not significant. Student’s *t-*test and two-way ANOVA followed by Tukey’s *post hoc*. Each dot represents an individual animal (*n* = 9–10 mice/group). Panel **A** created in BioRender. Grassi, C. (2026) https://BioRender.com/lcp5boi.

For this paradigm animals underwent two training sessions per day (5 min each, one in the morning and the second in the afternoon) for two consecutive days. The following day the animals underwent the interference session and 1 h later they performed the testing phase (Fig. 4A).

### Statistical analysis

Sample sizes were chosen with adequate statistical power (0.8) according to results of prior pilot data sets or studies, including our own, using similar methods or paradigms. Data was analysed using GraphPad Prism software. Data are expressed as mean ± standard error of the mean (SEM), with significance set at *P* < 0.05. Data were first tested for equal variance and normality (Shapiro–Wilk test). Behavioural results were analysed using unpaired two-tailed Student’s *t-*test or two-way analysis of variance (ANOVA) followed by Tukey’s *post hoc*.

## Results

### Female 3×tg-AD mice are vulnerable to RI

In our previous work, we had demonstrated that male WT mice are vulnerable to RI, whereas female WT are not.^19^ Therefore, we first aimed to determine whether this difference would be maintained in 2-month-old female and male 3×Tg-AD mice, an age at which no overt memory deficits are observed.^20^ To this end, mice were first subjected to a classic NOR paradigm, to assess baseline memory performance and, after 1-week washout, to the RI paradigm (Fig. 1A). In the standard NOR, both WT and 3×Tg-AD female and male mice showed normal memory, as the PI for the novel object was significantly higher compared to the PI of the old object for both groups (PI new versus PI old; female WT: 67.15 ± 4.12% versus 32.85 ± 4.12%, *P* < 0.001; Student’s *t-*test; female 3×Tg-AD: 67.28 ± 1.41% versus 32.72 ± 1.41%, *P* < 0.001; Student’s *t-*test, Fig. 1B; male WT: 65.66 ± 4.29% versus 34.34 ± 4.29%, *P* < 0.001; Student’s *t-*test; male 3×Tg-AD: 68.27 ± 3.72% versus 31.73 ± 3.72%, *P* < 0.001; Student’s *t-*test, Fig. 1D and E). When assessed using the NOR-RI paradigm, WT female mice were still able to discriminate the novel from the old object (PI new versus PI old: 66.37 ± 3.31% versus 33.64 ± 3.31%, *P* < 0.001; Student’s *t-*test, Fig. 1C). In contrast, female 3×Tg-AD mice failed to discriminate the novel from the old object (PI new versus PI old: 51.13 ± 2.96% versus 48.87 ± 2.96%, *P* = 0.596; Student’s *t-*test, Fig. 1C). Again, male WT failed to discriminate the novel from the old object, confirming previously published data and the same was observed for male 3×Tg-AD mice (male WT: 51.69 ± 1.44% versus 48.31 ± 1.44%, *P* > 0.05; Student’s *t-*test; male 3×Tg-AD: 51.31 ± 2.27% versus 48.69 ± 2.27%, *P* > 0.05; Student’s *t-*test, Fig. 1D and E). Finally, when directly comparing the performances of both sexes and genotype, we observed no differences in the standard NOR (DI: male, WT: 0.313 ± 0.043; male 3×Tg-AD: 0.365 ± 0.074; female WT: 0.346 ± 0.028; female 3×Tg-AD: 0.343 ± 0.082; *P* > 0.05 for all factors, two-way ANOVA, Fig. 1F). On the other hand, in the NOR-RI we observed significant differences for both genotype (*F*(1,34) = 7.870; *P* = 0.006), sex (*F*(1,34) = 7.920; *P* = 0.008) and interaction (*F*(1,34) = 8.343; *P* = 0.006). Tukey’s *post hoc* revealed that female WT had a significantly higher DI compared to all other conditions (male WT: 0.022 ± 0.034; female WT: 0.327 ± 0.069; male 3×Tg-AD: 0.027 ± 0.045; female 3×Tg-AD: 0.023 ± 0.062; female WT versus male WT *P* = 0.001; versus male 3×Tg-AD *P* = 0.001; versus female 3×Tg-AD *P* = 0.001, Fig. 1G). These data indicate that, while male mice of both strains show similar RI vulnerability, 3×Tg-AD female mice show increased vulnerability to RI, a phenotype observed at a prodromal phase, when overt cognitive deficits are absent. Of note, the oestrous cycle phase did not influence the performance of 3×Tg-AD mice in the NOR-RI (see Supplementaty Fig. 1A and B).

### 3×Tg-AD Mice and WT male mice are vulnerable to RI independently from timing of RI exposure.

Given the fact that, contrarily to WT females, both male WT and female and male 3×Tg-AD mice were RI vulnerable, we sought to determine whether RI vulnerability could depend on the precise timing of RI experience. Indeed, acquisition of novel information can disrupt memory if this happens during the consolidation phase or prior to memory retrieval.^21,22^ To this end, both WT and 3×Tg-AD male and female mice were repeatedly exposed to NOR-RI at different time points: (i) 1 h after training, corresponding to the consolidation phase; (ii) 6 h after training, that is outside the consolidation window; 23 h after training, that is 1 h before the retrieval, as a control. The test was performed 24 h after training (Fig. 2A). In the 1-h group, both female WT and 3×Tg-AD mice failed to discriminate the novel object (PI new versus PI old; WT: 53.10 ± 2.71% versus 46.90 ± 2.71%, *P* = 0.124; 3×Tg-AD: 48.59 ± 2.45% versus 51.41 ± 2.45%, *P* = 0.424; Student’s *t-*test, Fig. 2B and C), thus indicating that the consolidation phase is extremely sensitive to any form of interference. In the 6-h group, however, female WT mice were able to discriminate the novel from the old object, while female 3×Tg-AD mice failed to do so (PI new versus PI old; WT: 69.46 ± 3.15% versus 30.54 ± 3.15%, *P* < 0.001; 3×Tg-AD: 52.12 ± 3.73% versus 47.89 ± 3.73%, *P* = 0.433; Student’s *t-*test, Fig. 2B and C). In the 23-h group, as previously observed, female 3×Tg-AD mice failed to discriminate the novel from the old object (PI new versus PI old; 50.57 ± 3.08% versus 49.43 ± 3.08%, *P* = 0.798; Student’s *t-*test, Fig. 2C). Concerning male WT and 3×Tg-AD mice, they both failed to discriminate the novel object in all the conditions tested (1-h group; PI new versus PI old; WT: 46.34 ± 3.62% versus 53.66 ± 3.62%, *P* = 0.171; 3×Tg-AD: 45.73 ± 5.91% versus 54.27 ± 5.91%, *P* = 0.320; 6-h group; PI new versus PI old; WT: 50.26 ± 3.39% versus 49.74 ± 3.39%, *P* = 0.916; 3×Tg-AD: 45.96 ± 4.32% versus 54.04 ± 4.32%, *P* = 0.203; 23-h group; PI new versus PI old; WT: 48.09 ± 2.20% versus 51.91 ± 2.20%, *P* = 0.234; 3×Tg-AD: 52.19 ± 5.22% versus 47.81 ± 5.22%, *P* = 0.560; Student’s *t-*test, Fig. 2D and E). Finally, when comparing the performances of both sexes and strain, we did not observe a significant difference between WT and 3×Tg-AD mice (DI: male WT: −0.073 ± 0.072; male 3×Tg-AD: −0.085 ± 0.118; female WT: 0.062 ± 0.054; female 3×Tg-AD: −0.058 ± 0.046; *P* > 0.05 for all factors, two-way ANOVA, Fig. 2F) at the 1-h interval. On the other hand, we observed significant differences for both genotype (*F*(1,35) = 8.56; *P* = 0.006) and sex (*F*(1,35) = 11.75; *P* = 0.002) at the 6-h interval. Tukey’s *post hoc* revealed that female WT had a significantly higher DI compared to all other conditions (male WT: 0.005 ± 0.068; male 3×Tg-AD: −0.081 ± 0.086; female WT: 0.389 ± 0.063; female 3×Tg-AD: 0.042 ± 0.075; male WT versus female WT *P* = 0.005; male 3×Tg-AD versus female WT *P* < 0.001; female 3×Tg-AD versus female WT *P* = 0.01; two-way ANOVA, Tukey’s *post hoc*; Fig. 2G). Moreover, we observed significant differences for both sex (*F*(1,35) = 5.460; *P* = 0.025) and interaction (*F*(1,35) = 7.701; *P* = 0.009) at the 23-h interval. Tukey’s *post hoc* revealed that female WT had a significantly higher DI compared to all other conditions (male WT: −0.039 ± 0.044; male 3×Tg-AD: 0.044 ± 0.104; female WT: 0.340 ± 0.067; female 3×Tg-AD: 0.011 ± 0.062; male WT versus female WT *P* = 0.006; male 3×Tg-AD versus female WT *P* = 0.034; female 3×Tg-AD versus female WT *P* = 0.016; two-way ANOVA, Tukey’s *post hoc*; Fig. 2H). Collectively, these results suggest that, while the 1 h RI interval was deleterious to both strains and sexes, only WT female mice are resistant to the 6 and 23 h RI paradigm.

### Context regulates interference vulnerability in female 3×tg-AD and WT male mice but not in male 3×tg-AD mice

One possible explanation of vulnerability to RI may rely on the similarity between two memories: the more overlapping they are in terms of content, the harder it becomes to distinguish them.^19^ To evaluate the impact of cues on the efficacy of interference paradigm, we performed the NOR-RI paradigm in a different context (ctx) to differentiate the two memories (Fig. 3A). Concerning WT female mice, their performance showed normal recognition memory in all the conditions tested (standard NOR; PI new versus PI old; female WT: 69.78 ± 1.66% versus 30.22 ± 1.66%, *P* < 0.001; NOR-RI same ctx; PI new versus PI old; female WT: 66.36 ± 1.91% versus 33.64 ± 1.91%, *P* < 0.001; NOR-RI different ctx; PI new versus PI old; female WT: 65.56 ± 2.27% versus 34.44 ± 2.27%, *P* < 0.001; Student’s *t-*test, Fig. 3B). When exposed to NOR-RI in a different context, female 3×Tg-AD mice were able to discriminate between the novel and old object (PI new versus PI old; diff. ctx: 68.44 ± 2.85% versus 31.57 ± 2.85%, *P* < 0.001; Student’s *t-*test; Fig. 3C), thus suggesting that the similarity level of the two memories may influence RI vulnerability in 3×Tg-AD mice. Concerning male mice, when exposed to NOR-RI in a different context, male WT mice showed normal memory, unlike when they carried out NOR-RI in the same context (PI new versus PI old; diff. ctx: 72.94 ± 3.99% versus 27.08 ± 3.99%, *P* < 0.001; same ctx: 51.53 ± 2.98% versus 48.47 ± 2.98%, *P* = 0.478; Student’s *t-*test; Fig. 3D). On the other hand, male 3×Tg-AD mice showed impaired memory even if the NOR-RI was experienced in a different context (PI new versus PI old; same ctx: 48.94 ± 3.41% versus 51.06 ± 3.41%, *P* = 0.666; diff. ctx: 47.82 ± 3.39% versus 52.18 ± 3.39%, *P* = 0.374; Student’s *t-*test; Fig. 3E), thus suggesting that, in males, which are naturally more inclined to suffer RI, the combination of sex × genotype further aggravated the phenotypical impairment. When directly comparing the performances across sex and genotype, we observed no differences between groups in the standard NOR (DI: male WT: 0.303 ± 0.044; male 3×Tg-AD: 0.277 ± 0.044; female WT: 0.396 ± 0.033; female 3×Tg-AD: 0.356 ± 0.078; *P* > 0.05 for all factors, two-way ANOVA, Fig. 3F). On the other hand, we observed significant differences for both genotype (*F*(1,34) = 10.12; *P* = 0.003), sex (*F*(1,34) = 10.04; *P* = 0.003) and interaction (*F*(1,34) = 5.028; *P* = 0.032). Tukey’s *post hoc* revealed that female WT had a significantly higher DI compared to all other conditions (male WT: 0.031 ± 0.060; male 3×Tg-AD: −0.021 ± 0.068; female WT: 0.328 ± 0.038; female 3×Tg-AD: 0.030 ± 0.040; male WT versus female WT *P* = 0.004; male 3×Tg-AD versus female WT *P* < 0.001; female 3×Tg-AD versus female WT *P* = 0.004; two-way ANOVA, Tukey’s *post hoc*; Fig. 3G). In contrast, we observed significant differences for both genotype (*F*(1,33) = 11.38; *P* = 0.002) and interaction (*F*(1,33) = 18.02; *P* < 0.001) in the NOR-RI paradigm when tested in a different context. Tukey’s *post hoc* revealed that male 3×Tg-AD had a significantly lower DI compared to all other conditions (male WT: 0.459 ± 0.080; male 3×Tg-AD: −0.044 ± 0.068; female WT: 0.311 ± 0.045; female 3×Tg-AD: 0.369 ± 0.057; male WT versus male 3×Tg-AD *P* < 0.001; female WT versus male 3×Tg-AD *P* = 0.003; female 3×Tg-AD versus male 3×Tg-AD *P* < 0.001; two-way ANOVA, Tukey’s *post hoc*; Fig. 3H).

### Interference vulnerability is dependent on memory strength

Finally, we investigated whether memory strength could impact RI vulnerability. To this end, WT and 3×Tg-AD male and female mice underwent a multiple training sessions paradigm before being subjected to the RI protocol, as outlined in Fig. 4A. Again, as shown also in all the other paradigms, female WT showed normal recognition memory in both settings (single training NOR-RI; PI new versus PI old: 66.42 ± 2.98% versus 33.58 ± 2.98%, *P* < 0.001; multiple training NOR-RI; PI new versus PI old: 67.84 ± 3.48% versus 32.16 ± 3.48%, *P* < 0.001; Student’s *t-*test, Fig. 4B). After a multiple training sessions paradigm before the NOR-RI protocol, female 3×Tg-AD mice were able to discriminate the novel from the old object (PI new versus PI old; 74.37 ± 2.72% versus 25.64 ± 2.72%, *P* < 0.001; Student’s *t-*test, Fig. 4C). Male WT mice were also able to discriminate the novel from the old object in the test phase (PI new versus PI old; 73.06 ± 4.85% versus 26.94 ± 4.85%, *P* < 0.001; Student’s *t-*test, Fig. 4D), thus suggesting that increasing memory strength can effectively reduce the vulnerability to RI. Male 3×Tg-AD showed a significant preference for the novel object (PI new versus PI old; 56.56 ± 2.90% versus 43.43 ± 2.90%, *P* = 0.006; Student’s *t-*test, Fig. 4E). When comparing the DI across genotype and sex in the NOR-RI single training paradigm, we observed significant differences for both genotype (*F*(1,35) = 4.418; *P* = 0.043), sex (*F*(1,35) = 8.665; *P* = 0.006) and interaction (*F*(1,35) = 4.890; *P* = 0.034). Tukey’s *post hoc* revealed that female WT had a significantly higher DI compared to all other conditions (male WT: 0.008 ± 0.074; male 3×Tg-AD: 0.015 ± 0.057; female WT: 0.328 ± 0.060; female 3×Tg-AD: 0.060 ± 0.055; male WT versus female WT *P* = 0.005; male 3×Tg-AD versus female WT *P* = 0.006; female 3×Tg-AD versus female WT *P* = 0.024; two-way ANOVA, Tukey’s *post hoc*; Fig. 4F). Notably, when comparing the performance in multiple training NOR-RI, we observed a significant difference for interaction (*F*(1,34) = 10.01; *P* = 0.003). Tukey’s *post hoc* revealed that male 3×Tg-AD had a lower DI compared to both male WT and female 3×Tg-AD, but not compared to female WT (male WT: 0.461 ± 0.097; male 3×Tg-AD: 0.131 ± 0.058; female WT: 0.357 ± 0.070; female 3×Tg-AD: 0.487 ± 0.054; male WT versus male 3×Tg-AD *P* = 0.015; female 3×Tg-AD versus male 3×Tg-AD *P* = 0.008; female WT versus male 3×Tg-AD *P* = 0.162; two-way ANOVA, Tukey’s *post hoc*; Fig. 4G), thus suggesting that in male 3×Tg-AD mice, the impairment was still present, although to a lesser degree.

## Discussion

Identification of subtle cognitive vulnerabilities preceding overt memory deficits remains a critical challenge in Alzheimer’s disease research, as early detection of the pathology could enable earlier intervention and improve outcomes.^24^ Current guidelines for the diagnosis of Alzheimer’s disease include molecular biomarker analyses, neuroimaging and cognitive assessments. Aβ proteinopathy and hyperphosphorylated Tau at residues 217, 181 or 231, detected in cerebrospinal fluid, plasma or in the brain through positron emission tomography (PET) are considered as the *conditio sine qua non* for the diagnosis of Alzheimer’s disease.^25^ Cognitive assessment remains particularly challenging, given the heterogeneity of diseases that can induce cognitive impairment in old age. This variability makes it difficult to attribute a particular memory fault to a future diagnosis of Alzheimer’s disease, especially at the MCI stage, where memory deficits are present but are not severe enough to be classified as dementia.^26-36^ Clinically, cognitive assessment of MCI and Alzheimer’s disease patients is done using the MMSE or the MoCA which, however, do not assess the impact of interference on memory vulnerability.^37^

Recent evidence has begun to accumulate showing that vulnerability to interference, both proactive and retroactive, can be used to specifically assess MCI and Alzheimer’s disease progression. For instance, the work from Crocco *et al*. reported that assessing RI and proactive interference using the Loewenstein–Acevedo Scales of Semantic Interference and Learning (LASSI-L) in amnestic MCI patients grants higher sensitivity compared to psychological tests commonly employed.^6^ Another work demonstrated that interference vulnerability in the LASSI-L correlated with amyloid PET signal and progression of MCI severity after 1 year.^38^ Again, in another report, it has been documented that semantic intrusions in cognitive tests are correlated to the levels of plasma pTau 181 and amyloid PET imaging, thus indicating that nuanced cognitive tests can detect and unmask subtler phenotypes.^39^

In our previous work, we demonstrated that female WT mice were insensitive to RI through an oestrogen receptor α-dependent mechanism at the level of the hippocampus.^19^ In the present study we asked how far that resilience extends and found that it is absent in 3×Tg-AD females at an age when standard object recognition memory is still preserved. In the standard RI paradigm, WT females were the only group able to discriminate the novel object, while WT males, transgenic males and transgenic females did not differ from one another. The transgenic female phenotype is therefore more accurately described as the loss of a protection that WT females possess than as an emergent vulnerability, since these animals were not more impaired than males of either genotype. These findings suggest that interference paradigms can reveal memory dysfunction earlier than classical recognition tasks, and that the loss of a resilience mechanism may be detectable before any overt memory deficit becomes apparent.

We first evaluated the temporal profile of RI vulnerability. The aim of this temporal distinction was to define the potential memory processes underlying RI vulnerability, trying to differentiate between consolidation or retrieval impairments, memory traces overlapping or accelerated memory decay. When happening in the few hours post-training, new learning can interfere with the consolidation phase of the previously encoded memory.^21^ For this reason, we compared the effect of RI under three different time points: (i) consolidation (1 h after training); (ii) post-consolidation (6 h after training) and (iii) pre-retrieval (23 h after training). The choice of 6 h as a post-consolidation time point was based on the pharmacological definition of the consolidation window for object recognition memory: infusion of the protein synthesis inhibitor anisomycin into the dorsal CA1 impairs consolidation of long-term object recognition memory when delivered immediately or 180 min after training, but not when delivered 360 min after training.^22^ We note that this boundary was established in rats, and that consolidation is not a single discrete process, since later protein-synthesis-dependent phases have been described;^40^ the 6 h time point should therefore be understood as falling outside the early consolidation window rather than outside consolidation in an absolute sense. Female WT mice showed vulnerability to RI only when interference was experienced during consolidation, but not at the 6 and 23 h time points. RI vulnerability in female 3×Tg-AD mice was not restricted to a specific temporal window, as they remained impaired at all the time points during which interference occurred. WT and 3×Tg-AD males were likewise impaired at 1, 6 and 23 h. These results suggest that the disruption produced by interference delivered during consolidation is common to both sexes and both genotypes and is therefore unlikely to represent the substrate of the transgene-related phenotype. As it has been previously suggested, new learning during memory consolidation leads to competition for the plasticity-related resources engaged by the first learning episode.^41^

Concerning the classical RI paradigm employed here, which is represented by the 23 h interval, it is possible that interference may act at retrieval: as we have previously shown, the interfering object pair is itself retained by both males and females,^19^ and consolidation of this second memory could compete with retrieval of the first, leaving the original trace intact but inaccessible. However, this interpretation does not fit with the results obtained from the 6 h interval, as memory consolidation for the interfering couple should be closed by the time the test is performed. An alternative explanation may come from interference shortening the lifetime of the original trace. Object recognition memory acquired in a single session is retained for <72 h in mice, and its loss is accelerated by activation of the small GTPase Rac1, which is also engaged by RI.^42^ Rac1 activity is elevated in the hippocampus of Alzheimer’s disease patients and of Alzheimer’s disease mouse models, where it accelerates memory decay,^43^ offering a potential substrate for a genotype-dependent component of forgetting that is largely independent of the relationship between the two episodes. On this account, interference delivered at 6 h would reduce the lifespan of the original trace below the 24 h retention interval, without any requirement for competition at retrieval. Of note, accelerated decay could also be accompanied by RI causing memory overlap due to reduced pattern separation, leading to one memory overwriting the previous one.

To test the hypothesis of memory traces overlapping in regulating RI vulnerability, we employed a different RI paradigm, during which the context where RI is experienced is different from the original context, to create a sufficiently different memory trace. Context manipulation revealed that reducing similarity between memory traces rescues performance in female 3×Tg-AD mice, supporting the idea that, in females, interference vulnerability depends on representational overlap. This interpretation is consistent with the role of the dentate gyrus in separating similar representations, a function modulated by adult neurogenesis, as indicated by both experimental work and computational modelling of dentate gyrus circuitry,^44-46^ and impaired early in transgenic models of Alzheimer’s disease.^47^ Interestingly, this effect was absent in male 3×Tg-AD mice, which remained impaired even when contextual cues were dissociated. Importantly, this cannot be attributed to a general male insensitivity to contextual information: WT males, who are themselves vulnerable to RI when interference occurs in the same context,^19^ were fully rescued by context change, and their DI under this condition did not differ from that of transgenic females. Contextual separation is therefore an effective protective route in males, intact in WT animals but ineffective in transgenic ones.

Finally, we assessed whether variations in memory strength obtained through multiple training sessions could reduce vulnerability to interference. Accordingly, repeated memory reactivations reduce forgetting rates.^48,49^ When subjected to multiple training, female 3×Tg-AD mice showed no memory deficits in the test phase. Male 3×Tg-AD mice showed a statistically significant but quantitatively limited improvement, expressing a preference for the novel object while retaining a DI significantly lower than that of WT males and of transgenic females.

Taken together, these dissociations suggest that RI-induced forgetting in our paradigms reflects the contribution of two processes rather than one, weighted differently across sex and genotype. WT females are protected whenever interference falls outside the consolidation window. WT males and transgenic females are vulnerable to interference delivered in the same context at any time point yet recover both when representational overlap is reduced and when the original memory is strengthened, which is what would be expected if their deficit arose largely from competition between similar representations. Transgenic males are not rescued by contextual separation and only partially by memory strengthening, which points instead to a component of forgetting that is largely independent of the relationship between the two episodes. We emphasize that these are inferences drawn from the pattern of rescue rather than direct demonstrations and that the two processes are not mutually exclusive. Besides Alzheimer’s disease-related pathological mechanisms, therefore, sex-specific vulnerability should be considered when assessing RI susceptibility, as these sex-specific effects may relate to both hormonal differences and differences in connectivity and functioning within the brain areas involved.

Our previous identification of an ERα- and ERK1/2-dependent mechanism of female resilience^19^ raises the question of whether its disruption underlies the phenotype described here. Oestrous cycle staging in 3×Tg-AD mice showed no differences in high oestradiol versus low oestradiol animals in the NOR-RI, thus suggesting that the effect may be ascribed to altered oestradiol signalling and not oestradiol availability. Indeed, it has been shown that oestradiol levels in the brain tissue of 3×Tg-AD mice do not change with ageing compared to their WT counterparts.^50^ Another potential interpretation of the present data is that early AD-related pathology prevents the recruitment of this pathway in response to interference, and the potential levels at which it could fail include reduced ERα expression or availability, altered subcellular localization, impaired coupling to ERK1/2, and reduced local synthesis of oestradiol in the hippocampus. For instance, previous report has shown that ERα expression is reduced in the medial amygdala of 3×Tg-AD mice^50^ and that, in human Alzheimer’s disease brain samples, tau interacts aberrantly with ERα leading to its dysfunction.^51^ Furthermore, *in vitro* overexpression of tau in M17 cells leads to reduced ERα transcriptional activity upon oestradiol stimulation.^52^ However, although compelling, we cannot state that the rescue operated by context switching for RI triggers the correct activation of the ERα–ERK1/2 as WT males, in which this route is not operative, were rescued to the same extent. Contextual separation therefore may protect memory through a mechanism parallel to, rather than dependent on, the one we previously described.

Several limitations of our study should be considered. The present study focuses on behavioural end-points and does not directly address the underlying synaptic or molecular correlates of RI vulnerability. Future studies integrating electrophysiological, structural and biochemical analyses will be necessary to determine whether enhanced interference susceptibility reflects synaptic instability, altered hormonal signalling, or impaired hippocampal-cortical communication. As discussed above, our design also does not establish whether the original memory is degraded or rendered inaccessible by interference, since both effective manipulations were preventive rather than restorative.

No minimum exploration criterion was pre-specified in our original design. Total object exploration at test was low in male 3×Tg-AD mice in the multiple-training paradigm, in which exploration declined progressively across successive training sessions in all groups, consistent with habituation to repeated exposure to the same objects rather than with a genotype-specific effect. Although the preference and discrimination indices used here are relative measures, which correct for differences in exploratory activity and are consequently less sensitive to exploration bias than absolute measures, a minimum amount of exploration is nonetheless required for stable discrimination performance.^53^ The partial improvement observed in transgenic males after multiple training should therefore be interpreted with corresponding caution.

Finally, our conclusions derive from a mouse model, and no human validation cohort was examined here. Longitudinal studies will be required to establish whether early RI vulnerability predicts later cognitive decline in transgenic models, along with core markers employed to diagnose and classify Alzheimer’s disease stage. Overall, our findings identify the loss of resilience to RI as a sensitive and sex-dependent behavioural phenotype in 3×Tg-AD mice. By revealing memory fragility in the absence of overt deficits, RI paradigms may offer a valuable experimental and potentially translational tool for the early detection of Alzheimer’s disease-related cognitive decline.

## Supplementary Material

fcag345_Supplementary_Data

## Data Availability

Datasets employed in this work are available on request to the corresponding author.
