# Supplementary material for: Differences in retroactive interference response reveal sex-specific memory resilience in pre-symptomatic triple transgenic Alzheimer’s disease mice
Source: Brain Commun. 2026 Sep 11;8(5):fcag345. doi: 10.1093/braincomms/fcag345 (PMC13590231; doi:10.1093/braincomms/fcag345)
Supplement: Supplementary file 1 [file fcag345_supplementary_data.pdf]

Group	Training	Interference	Test
Female WT (Standard NOR)	21.51 ± 3.74 s	not applicable	16.59 ± 2.42 s
Female WT (NOR-RI)	22.35 ± 1.62 s	15.01 ± 1.91 s	11.65 ± 2.27 s
Female 3×Tg-AD (Standard NOR)	10.10 ± 1.46 s	not applicable	17.41 ± 1.98 s
Female 3×Tg-AD (NOR-RI)	15.48 ± 1.90 s	15.04 ± 1.95 s	10.92 ± 1.60 s
Male WT (Standard NOR)	16.98 ± 2.04 s	not applicable	17.34 ± 2.16 s
Male WT (NOR-RI)	22.00 ± 3.26 s	20.68 ± 1.45 s	21.87 ± 5.11 s
Male 3×Tg-AD (Standard NOR)	13.34 ± 1.18 s	not applicable	18.32 ± 2.16 s
Male 3×Tg-AD (NOR-RI)	15.43 ± 1.65 s	15.86 ± 1.62 s	18.80 ± 2.14 s
Female WT (NOR-RI 23 h)	13.99 ± 0.95 s	14.09 ± 1.28 s	18.16 ± 1.65 s
Female WT (NOR-RI 6 h)	17.83 ± 1.65 s	15.37 ± 0.60 s	19.58 ± 0.88 s
Female WT (NOR-RI 1 h)	12.64 ± 1.01 s	12.92 ± 1.34 s	13.96 ± 0.98 s
Female 3×Tg-AD (NOR-RI 23 h)	10.81 ± 1.32 s	8.89 ± 1.56 s	13.25 ± 1.23 s
Female 3×Tg-AD (NOR-RI 6 h)	10.96 ± 1.85 s	11.34 ± 2.20 s	8.42 ± 1.40 s
Female 3×Tg-AD (NOR-RI 1 h)	9.15 ± 1.26 s	9.79 ± 1.12 s	14.69 ± 0.76 s
Male WT (NOR-RI 23 h)	19.13 ± 2.70 s	22.82 ± 2.21 s	21.20 ± 1.76 s
Male WT (NOR-RI 6 h)	23.70 ± 3.30 s	18.86 ± 3.16 s	12.02 ± 1.52 s
Male WT (NOR-RI 1 h)	21.31 ± 1.96 s	27.04 ± 2.69 s	13.74 ± 2.23 s
Male 3×Tg-AD (NOR-RI 23 h)	5.56 ± 0.91 s	5.62 ± 0.74 s	7.32 ± 1.34 s
Male 3×Tg-AD (NOR-RI 6 h)	15.38 ± 3.07 s	6.60 ± 1.15 s	6.20 ± 1.35 s
Male 3×Tg-AD (NOR-RI 1 h)	8.98 ± 1.42 s	12.88 ± 2.30 s	4.62 ± 1.30 s
Female WT (Standard NOR)	36.53 ± 3.02 s	not applicable	23.22 ± 3.10 s
Female WT (NOR-RI same ctx)	12.40 ± 2.54 s	11.94 ± 2.13 s	14.18 ± 2.18 s
Female WT (NOR-RI diff. ctx)	16.99 ± 2.10 s	11.34 ± 1.90 s	13.31 ± 2.66 s
Female 3×Tg-AD (Standard NOR)	23.25 ± 3.27 s	not applicable	10.94 ± 1.50 s
Female 3×Tg-AD (NOR-RI same ctx)	25.59 ± 3.02 s	23.88 ± 3.17 s	22.70 ± 3.85 s
Female 3×Tg-AD (NOR-RI diff. ctx)	25.44 ± 2.31 s	20.26 ± 2.80 s	12.53 ± 1.39 s
Male WT (Standard NOR)	25.53 ± 1.48 s	not applicable	31.23 ± 3.18 s
Male WT (NOR-RI same ctx)	19.50 ± 3.51 s	18.58 ± 2.39 s	18.94 ± 2.10 s
Male WT (NOR-RI diff. ctx)	14.06 ± 1.37 s	12.55 ± 1.55 s	16.82 ± 1.54 s
Male 3×Tg-AD (Standard NOR)	25.66 ± 2.26 s	not applicable	29.16 ± 3.58 s
Male 3×Tg-AD (NOR-RI same ctx)	13.24 ± 2.11 s	8.15 ± 1.70 s	10.61 ± 1.77 s
Male 3×Tg-AD (NOR-RI diff. ctx)	5.38 ± 1.71 s	10.33 ± 2.80 s	9.67 ± 2.90 s
Female WT (NOR-RI single training)	15.60 ± 1.68 s	17.24 ± 2.01 s	14.09 ± 1.73 s
Female WT (NOR-RI multiple training)	23.98 ± 3.60 s<br>17.89 ± 3.74 s<br>17.69 ± 2.24 s<br>13.84 ± 2.64 s	24.07 ± 2.27 s	13.12 ± 1.88 s
Female 3×Tg-AD (NOR-RI single training)	13.83 ± 1.81 s	10.93 ± 1.81 s	14.97 ± 2.56 s
Female 3×Tg-AD (NOR-RI multiple training)	11.92 ± 1.58 s<br>15.00 ± 2.75 s<br>12.72 ± 2.36 s<br>10.93 ± 1.30 s	16.67 ± 1.98 s	11.17 ± 1.51 s
Male WT (NOR-RI single training)	16.44 ± 1.72 s	18.23 ± 2.41 s	19.82 ± 1.65 s
Male WT (NOR-RI multiple training)	17.77 ± 1.45 s<br>10.59 ± 1.13 s<br>8.42 ± 0.49 s<br>5.42 ± 0.82 s	16.50 ± 1.66 s	10.82 ± 1.23 s
Male 3×Tg-AD (NOR-RI single training)	24.43 ± 2.96 s	19.14 ± 2.29 s	15.85 ± 1.35 s
Male 3×Tg-AD (NOR-RI multiple training)	11.41 ± 2.36 s<br>9.94 ± 1.63 s<br>8.18 ± 1.43 s<br>6.12 ± 0.96 s	8.61 ± 1.70 s	6.44 ± 0.82 s

Supplementary Table 1. Exploration time for all phases.

Supplementary information

Protocol for oestrous cycle monitoring.

For oestrous cycle monitoring, the procedure was performed as described in [1]. Briefly, after the NOR-RI test, the animals were gently restrained by the experimenter, and the vaginal wash was performed using 20 µL of PBS. The collected fluid was then smeared onto a clean glass microscope slide and allowed to air-dry at room temperature. Samples were examined unstained under a light microscope at 10× magnification, and images were acquired for subsequent classification. The oestrous cycle stage was determined on the basis of the relative proportions of the three main cell types, namely nucleated epithelial cells, cornified squamous epithelial cells and leukocytes: proestrus was defined by a predominance of nucleated epithelial cells, oestrus by clustered anucleated cornified cells, metoestrus by a mixture of cornified cells and leukocytes, and dioestrus by a predominance of leukocytes.

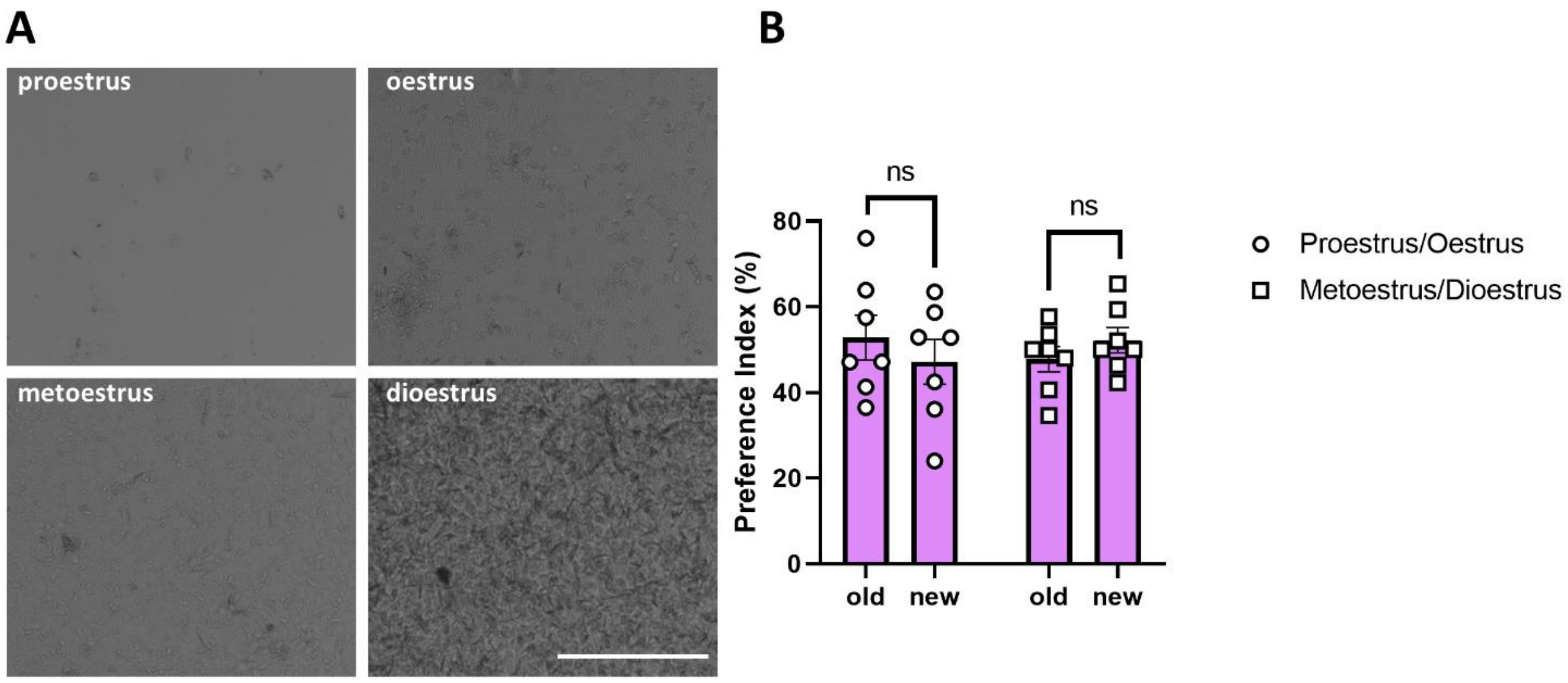

Supplementary Figure 1. Phases of the oestrous cycle do not influence RI vulnerability in 3×Tg-AD mice. (**A**) Representative images of vaginal fluid collected during different oestrous cycle phases. (**B**) PI for 3×Tg-AD females undergoing NOR-RI under different oestrous cycle phases. Each point represents the PI for the new object of one animal and the PI of the old object of the same animal. The left side of bar graph (round dots) represents animals in proestrus/oestrus, while the right side (square dots) represents animals in metoestrus/dioestrus. PI for New vs. Old: Proestrus/Oestrus: 52.78±5.63% vs. 47.21±5.63%; $p>0.05$, Student's t test; Metoestrus/Dioestrus: 47.8±3.19% vs. 52.19±3.19%, $p>0.05$, Student's t test. Data are reported as mean ± SEM. Scale bar: 200 µm.

Bibliography

1. Caligioni CS. Assessing reproductive status/stages in mice. Curr Protoc Neurosci. 2009;Appendix 4:Appendix-4I. doi:10.1002/0471142301.nsa04is48
